# Combined administration of anisodamine and neostigmine rescued acute lethal crush syndrome through α7nAChR-dependent JAK2-STAT3 signaling

**DOI:** 10.1038/srep37709

**Published:** 2016-11-22

**Authors:** Zhe-Qi Xu, Bo-Zong Shao, Ping Ke, Jian-Guo Liu, Guo-Ku Liu, Xiong-Wen Chen, Ding-Feng Su, Chong Liu

**Affiliations:** 1 Department of Pharmacology, School of Pharmacy, Second Military Medical University, Shanghai, China; 2 Cardiovascular Research Center, Temple University School of Medicine, Philadelphia, PA 19140, USA

## Abstract

Previously we showed that Ani (anisodamine)/Neo (neostigmine) combination produced anti-shock effect via activating α7 nicotinic acetylcholine receptor (α7nAChR). In this study, we aim to investigate the therapeutic effect and underlying mechanisms of Ani/Neo combination in acute lethal crush syndrome (CS). In rat and rabbit CS models, Ani/Neo combination increased the 24 h survival rates, improved hemodynamics and decreased the levels of creatine kinase, MB isoenzyme of creatine kinase, blood urea nitrogen, creatinine, K^+^ in serum. It also decreased the levels of H_2_O_2_, myeloperoxidase (MPO) and nitric oxide (NO) in serum and compressed muscle in rat CS model. In wild-type (WT) mice with CS, Ani/Neo combination increased 24 h survival rate and decreased the levels of H_2_O_2_, MPO, NO, TNFα, IL-6 and IL-10 in compressed muscle. These effects were attenuated by α7nAChR knockout (KO). Moreover, Ani/Neo combination prevented the decrease of phosphorylation of Janus kinase 2 (JAK2) and phosphorylation of signal transducer and activator of transcription 3 (STAT3) induced by CS. These effects of Ani/Neo in CS mice were cancelled by methyllycaconitine (α7nAChR antagonist) and α7nAChR KO. Collectively, our results demonstrate that Ani/Neo combination could produce therapeutic effects in CS. The underlying mechanism involves the activation of α7nAChR-dependent JAK2-STAT3 signaling pathway.

Crush syndrome (CS) is a serious medical condition which frequently occurs in catastrophic events such as earthquakes, debris flows, landslides, stampedes and vehicle accidents. CS is characterized by hyperkalemia, metabolic acidosis, hypovolemic shock, myoglobinuria, and acute kidney failure^1^,^2^. Currently, recommended treatment for CS involves early fluid replacement using physiological saline, basification of urine using bicarbonate, diuresis using mannitol and correction of hyperkalemia using calcium gluconate and glucose-insulin infusion^3^,^4^. Acute kidney failure is the most fatal outcome for CS patients, since it may lead to refractory hyperkalemia, acidosis, and uremia. In such cases, the transfer of the patient to a hospital for hemodialysis is urgently needed. At present, CS still has a high mortality with the majority of patients being dead soon after decompression. The most important pathophysiologic mechanism accounting for the high mortality is ischemia/reperfusion (I/R) induced rhabdomyolysis and subsequent systemic inflammation mediated oxidative stress^4^,^5^. Current recommended treatments only provide symptomatic management. To save the lives of CS patients immediately after decompression is still a medical challenge.

The central nervous system (CNS) may inhibit the secretion of proinflammatory cytokines from activated macrophages through the release of acetylcholine from the efferent vagus nerve terminals and subsequent activation of α7 nicotinic acetylcholine receptors (α7nAChR) on the macrophages. This is termed cholinergic anti-inflammatory pathway^6^,^7^,^8^. We have shown that anisodamine (Ani) promotes the binding of α7nAChR to endogenous acetylcholine and blocks muscarinic receptors^9^,^10^. Combining Ani with neostigmine (Neo), a cholinesterase inhibitor to increase endogenous acetylcholine, significantly augments the anti-shock effect in a dog hemorrhagic shock model and a murine endotoxic shock model through an α7nAChR-dependent mechanism^9^,^11^. Based on these studies, we hypothesize that combined Ani and Neo may produce therapeutic benefits for CS via activation of α7nAChR. We, to test this hypothesis, performed a series of experiment with rat, rabbit, and mouse CS models.

## Results

### Combined Ani and Neo increases the 24 h survival of rats with CS

**Figure 1:**
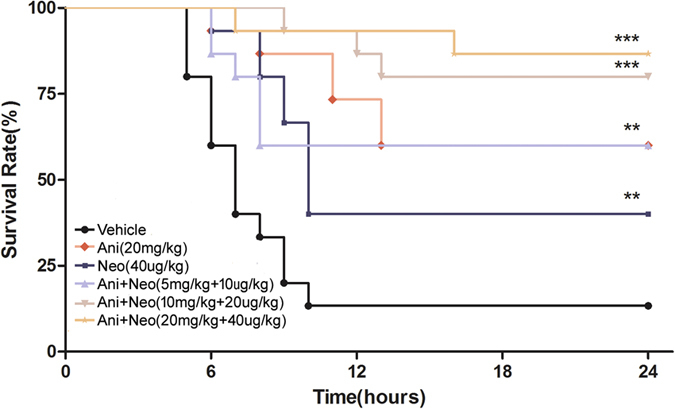
Effects of Ani and Neo on 24 h survival rate in rat CS model. Rats were subjected to hind-limbs compression for 5 h, followed by treatment with vehicle, Ani (20 mg/kg, i.p.), Neo (40 μg/kg, i.p.), or Ani/Neo combination at three different doses (5 mg/kg and 10 μg/kg, 10 mg/kg and 20 μg/kg, 20 mg/kg and 40 μg/kg, i.p.) at 30 min before decompression. Survival rates were monitored for the ensuing 24 h. n = 15 per group. *P < 0.05 *vs*. vehicle, **P < 0.01 *vs*. vehicle, ***P < 0.001 *vs*. vehicle. Ani, anisodamine; Neo, neostigmine.

We first determined the best combination of Ani (0, 5, 10, 20 mg/kg, i.p.) and Neo (0, 5, 10, 20, 40 μg/kg, i.p.) on the 24 h survival of rats subjected to CS. The 24 h survival rate in vehicle-treated CS group is 13.3%, which was the worst of all treatments. Ani alone (5, 10, and 20 mg/kg) improved the 24 h survival rate to 33.3%, 46.7%, 60%, respectively. Neo alone (5, 10, 20 μg/kg) also augmented the 24 h survival rate to 20%, 33.3% and 40%, respectively. The effects of the combinations of these two drugs were better than the effects of either drug at corresponding dose alone. Among the combined treatments, the combination of 10 mg/kg Ani and 20 μg/kg Neo increased the 24 h survival rate to 80% (Table1). The combined treatment of 20 mg/kg Ani and 40 μg/kg Neo further augmented the 24 h survival rate to 86.7% (Table 1 and Fig. 1). Therefore, we made a compound containing 20 mg/kg Ani and 40 μg/kg Neo (the Ani/Neo compound) for treating rat CS in the following experiments.

**Figure 2:**
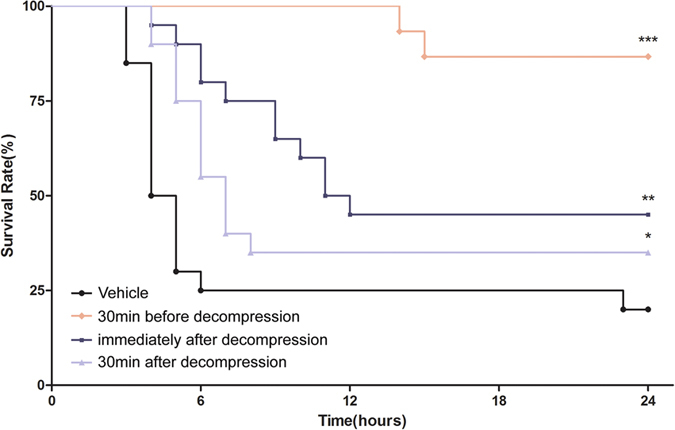
The optimal time for administration of the Ani/Neo compound in rat CS model. Rats were subjected to hind-limbs compression for 5 h, followed by treatment with vehicle, or the Ani/Neo compound (20 mg/kg and 40 μg/kg, i.p.) at 30 min before decompression, immediately after decompression and 30 min after decompression respectively. Survival rates were monitored for the ensuing 24 h. n = 20 per group. *P < 0.05 *vs*. vehicle, **P < 0.01 *vs*. vehicle, ***P < 0.001 *vs*. vehicle. Ani, anisodamine; Neo, neostigmine.

In the experiment for determining the optimal time for the administration of the Ani/Neo compound (20 mg/kg Ani and 40 μg/kg Neo), the compound given at 30 min before decompression, immediately after decompression and 30 min after decompression increased the survival rate to 86.7%, 45%, 35%, respectively (Fig. 2). According to these results, the optimal time for drug administration was at 30 min before decompression and this treatment modality was used for the following experiments.

### Effects of the Ani/Neo compound on mean arterial pressure (MAP), systolic blood pressure (SBP) and heart rate of rats with CS

**Figure 3:**
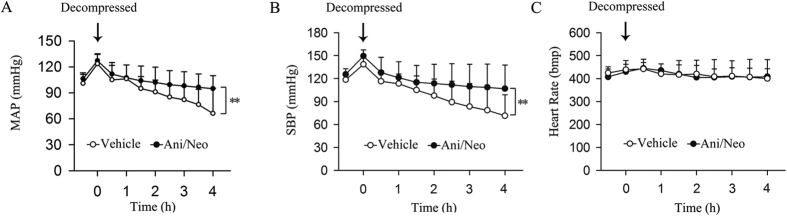
Effects of the Ani/Neo compound on MAP, SBP and heart rate in rat CS model. Rats were subjected to hind-limbs compression for 5 h, followed by treatment with vehicle or the Ani/Neo compound (20 mg/kg and 40 μg/kg, i.p.) at 30 min before decompression. Changes in MAP (**A**), SBP (**B**) and heart rate (**C**) in the first 4 h after decompression. n = 20 per group. **P < 0.01 *vs*. vehicle. Ani, anisodamine; Neo, neostigmine.

Before decompression, there is no difference in MAP and SBP of CS rats between vehicle group and the Ani/Neo compound group. After decompression, pretreatment with the Ani/Neo compound significantly improved MAP within 4 h compared with the vehicle group (from 127.27 ± 8.51 mmHg to 94.90 ± 15.15 mmHg in the Ani/Neo compound group *vs*. from 123.70 ± 11.34 mmHg to 66.07 ± 29.39 mmHg in vehicle group) (Fig. 3A). Consistently, SBP also was improved (from 149.40 ± 8.10 mmHg to 114.25 ± 22.50 mmHg in the Ani/Neo compound group *vs*. from 140.10 ± 10.70 mmHg to 82.80 ± 23.60 mmHg in vehicle group) within 4 h after decompression by the Ani/Neo compound treatment (Fig. 3B). However, the Ani/Neo compound treatment had no effect on heart rate of rats with CS (Fig. 3C).

### Effects of combined Ani and Neo at 500:1 ratio on creatine kinase (CK), MB isoenzyme of creatine kinase (CK-MB), blood urea nitrogen (BUN), creatinine (Cr) and electrolyte in serum of rats with CS

Crush injury increased the levels of CK, CK-MB, BUN and Cr in serum in rats. Ani/Neo combination dose-dependently decreased the levels of CK, CK-MB, BUN and Cr in serum (Table 2). Moreover, crush injury significantly increased K^+^ in serum (Table 2), but had no effect on Na^+^ and Cl^−^ (data not shown). Treatment with Ani/Neo combination also dose-dependently decreased the level of K^+^ in serum (Table 2).

### Effects of the Ani/Neo compound on H_2_O_2_, myeloperoxidase (MPO) and nitric oxide (NO) in serum and compressed muscle of rats with CS

**Figure 4:**
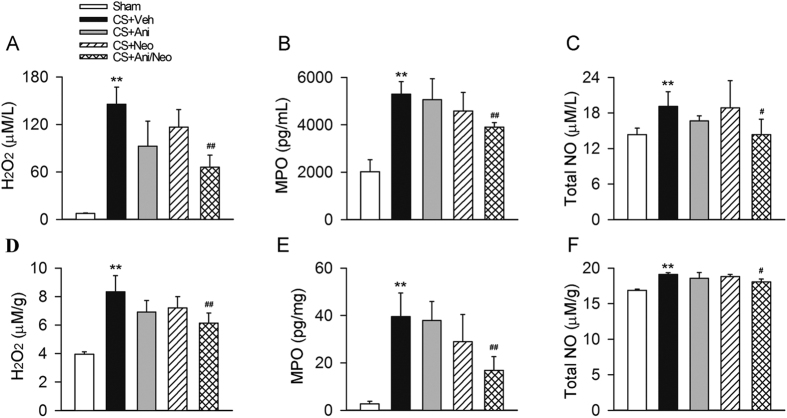
Effects of the Ani/Neo compound on levels of H_2_O_2_, MPO, and total NO in serum and compressed muscle in rat CS model. Rats were subjected to hind-limbs compression for 5 h, followed by treatment with physiological saline, Ani (20 mg/kg, i.p.); Neo (40 μg/kg, i.p.) or the Ani/Neo compound (20 mg/kg and 40 μg/kg, i.p.) at 30 min before decompression. Blood samples and compressed muscle were collected at 6 h after decompression. Levels of H_2_O_2_, MPO, and total NO in serum (**A–C**) and compressed muscle (**D–F**) were measured. n = 10 per group. Veh, vehicle; Ani, anisodamine; Neo, neostigmine; MPO, myeloperoxidase. **P < 0.01 *vs*. Sham, ^#^P < 0.05 *vs*. CS+Veh, ^##^P < 0.01 *vs*. CS+Veh.

Crush injury increased the levels of H_2_O_2_, MPO and total NO in serum and compressed muscle. Treatment with the Ani/Neo compound significantly decreased the levels of H_2_O_2_, MPO and total NO in serum (Fig. 4A–C) and compressed muscle (Fig. 4D–F) compared with vehicle group in rats with CS.

### Effects of combined Ani and Neo at 500:1 ratio on 24 h survival rate of rabbits with CS

**Figure 5:**
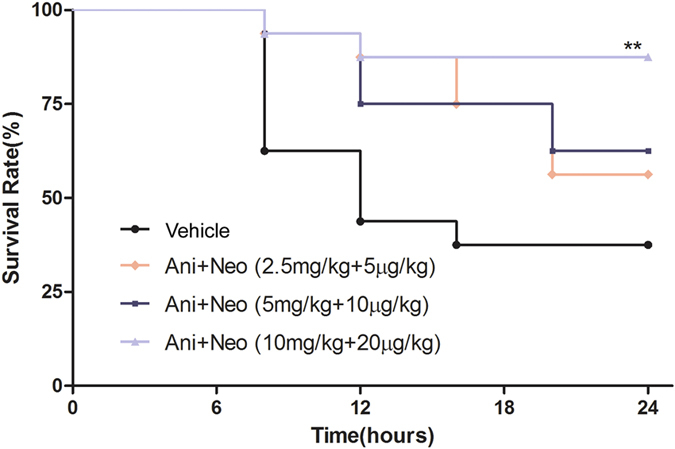
Effects of Ani/Neo combination on 24 h survival rate in rabbit CS model. Rabbits were subjected to hind-limbs compression for 6 h, followed by treatment with vehicle, or three different doses of Ani/Neo combination (2.5 mg/kg and 5 μg/kg, 5 mg/kg and 10 μg/kg, 10 mg/kg and 20 μg/kg, i.p.) at 30 min before decompression. Survival rates were monitored for the ensuing 24 h. n = 16 per group. Ani, anisodamine; Neo, neostigmine. **P < 0.01 *vs*. Vehicle.

Crush injury induced a decrease of 24 h survival rate to 37.5% in rabbits. Treatment with combined Ani and Neo with a ratio of 500:1 increased the 24 h survival rate to 56.3%, 62.5%, 87.5%, respectively (Fig. 5). The best protective dose of combining treatment in rabbits with CS is the combination of 10 mg/kg Ani and 20 μg/kg Neo, which is equivalent the combination of 20 mg/kg Ani and 40 μg/kg Neo in rats with CS considering the body surface area.

### Effects of combined Ani and Neo at 500:1 ratio on CK, CK-MB, BUN, Cr and K^+^ in serum in rabbits with CS

Crush injury increased the level of CK, CK-MB, BUN and Cr in serum in rabbits. The combination of 2.5 mg/kg Ani and 5 μg/kg Neo showed no effect on CK, CK-MB, BUN, Cr in serum in CS rabbits. Combining 5 mg/kg Ani with 10 μg/kg Neo significantly decreased the level of CK and CK-MB, but had no effect on BUN and Cr in serum. A high dose of combination of 10 mg/kg Ani and 20 μg/kg Neo decreased the levels serum of CK, CK-MB, BUN and Cr in rabbits (Table 3). Moreover, crush injury significantly increased K^+^ level. Treated with Ani/Neo combination dose-dependently decreased the level of K^+^ in serum in rabbits (Table 3).

### Effects of combined Ani and Neo at 500:1 ratio on 24 h survival rate in wild-type (WT) and α7nAChR knockout (KO) mice with CS

**Figure 6:**
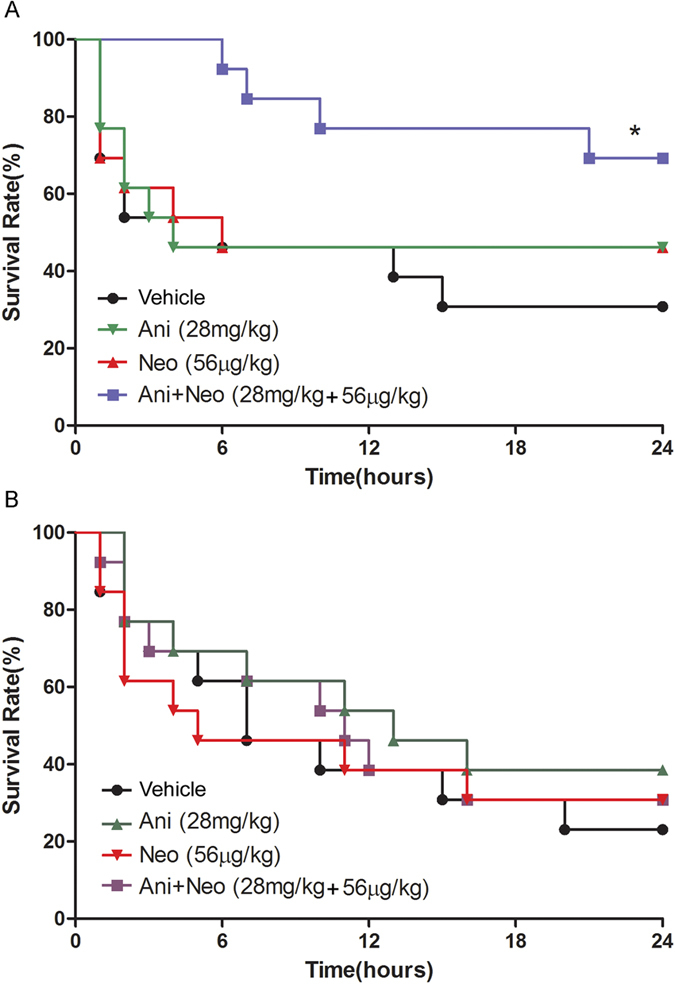
Effects of Ani/Neo combination on 24 h survival rate in WT and α7nAChR KO mice CS model. (**A**) WT and (**B**) α7nAChR KO mice were subjected to hind-limbs compression for 4 h, followed by treatment with vehicle; Ani (28 mg/kg, i.p.); Neo (56 μg/kg, i.p.) or Ani/Neo combination (28 mg/kg and 56 μg/kg, i.p.) at 30 min before decompression. Survival rates were monitored for the ensuing 24 h. WT, wild-type; KO, knockout; Ani, anisodamine; Neo, neostigmine. *P < 0.05 *vs*. Veh.

For mice experiments, we considered the combination of 28 mg/kg Ani and 56 μg/kg Neo as the best potential protective dose when normalizing to the body surface area of the mice. In WT mice, Ani/Neo combination treatment significantly increased the 24 h survival rate compared with vehicle group (69.2% *vs*. 30.8%, P < 0.05, Fig. 6A). In α7nAChR KO mice, the combined treatment did not improve survival rate compared with vehicle (30.8% *vs*. 23.1%, P > 0.05, Fig. 6B).

### Effects of combined Ani and Neo at 500:1 ratio on H_2_O_2_, MPO, NO, TNFα, IL-6 and IL-10 in compressed muscle in WT and α7nAChR KO mice with CS

**Figure 7:**
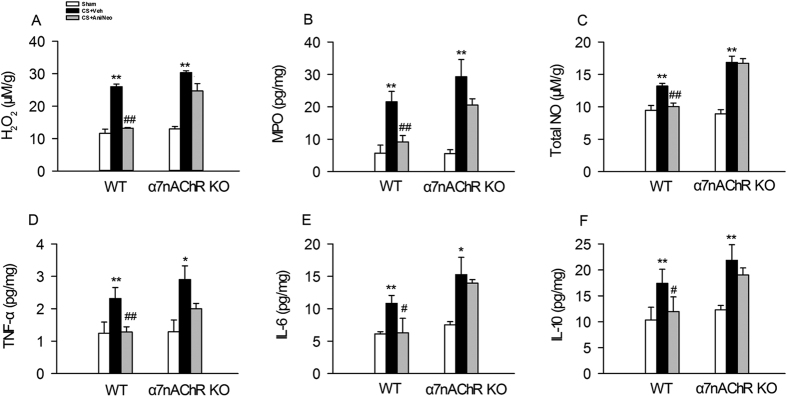
Effects of Ani/Neo combination on levels of H_2_O_2_, MPO, total NO, TNFα, IL-6, and IL-10 in decompressed muscle in WT and α7nAChR KO mice CS model. WT and α7nAChR KO mice were subjected to hind-limbs compression for 4 h, and then injected with vehicle; or Ani/Neo combination (28 mg/kg and 56 μg/kg, i.p.) at 30 min before decompression. Compressed muscle was collected at 6 h after decompression. Levels of H_2_O_2_, MPO, total NO, TNFα, IL-6, and IL-10 in compressed muscle were measured respectively. n = 6 per group. Veh, vehicle; MPO, myeloperoxidase; WT, wild-type; KO, knockout; Ani, anisodamine; Neo, neostigmine; *P < 0.05 *vs*. Sham, **P < 0.01 *vs*. Sham, ^#^P < 0.05 *vs*. CS+Veh, ^##^P < 0.01 *vs*. CS+Veh.

Crush injury increased the levels of H_2_O_2_, MPO and total NO in compressed muscle in both WT and α7nAChR KO mice. In WT mice with CS, the combined therapy (28 mg/kg Ani and 56 μg/kg Neo, i.p.) significantly decreased the levels of H_2_O_2_, MPO and total NO in compressed muscle compared with vehicle (Fig. 7A–C). While in α7nAChR KO mice with CS, treatment with Ani/Neo combination did not decrease the levels of H_2_O_2_, MPO and total NO.

Moreover, crush injury increased TNFα, IL-6 and IL-10 in compressed muscle in both WT and α7nAChR KO mice. Ani/Neo combination treatment significantly decreased the levels of TNFα, IL-6 and IL-10 in compressed muscle compared with vehicle in WT mice with CS. While in α7nAChR KO mice with CS, treatment with Ani/Neo combination had no effect compared with vehicle group (Fig. 7D–F).

### Effects of the combined Ani and Neo at 500:1 ratio on the phosphorylation of JAK2 and STAT3 in the compressed muscle tissue from C57BL/6 mice, WT and α7nAChR KO mice with CS

**Figure 8:**
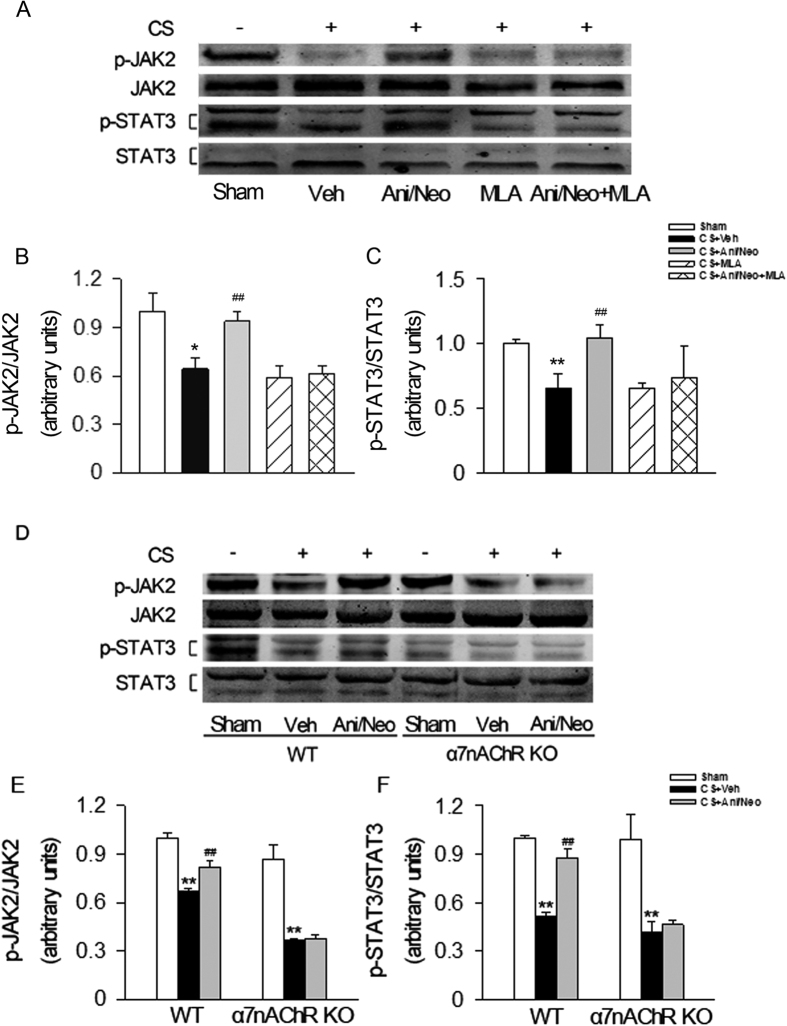
JAK2-STAT3 signaling pathways were involved in the protective effects of Ani/Neo combination on CS model. (**A–C**) C57BL/6 mice were subjected to hind-limbs compression for 4 h and then were intraperitoneally injected with vehicle; Ani/Neo combination (28 mg/kg and 56 μg/kg, i.p.); MLA (10 mg/kg, i.p.); Ani/Neo combination (28 mg/kg and 56 μg/kg, i.p.) + MLA (10 mg/kg, i.p.). Compressed muscles were collected at 6 h after decompression and levels of p-JAK2, JAK2, p-STAT3 and STAT3 were detected using Western blotting. n = 6 per group. Veh, vehicle; Ani, anisodamine; Neo, neostigmine; CS, crush syndrome; MLA, methyllycaconitine. *P < 0.05 *vs*. Sham, **P < 0.01 *vs*. Sham, ^##^P < 0.01 *vs*. CS + Veh. (**D–F**) WT and α7nAChR KO mice were subjected to hind-limbs compression for 4 h, and then followed by vehicle or Ani/Neo combination (28 mg/kg and 56 μg/kg, i.p.) 30 min before decompression. Compressed muscles were collected at 6 h after decompression and the levels of p-JAK2, JAK2, p-STAT3 and STAT3 were detected using Western blotting. n = 6 per group. Veh, vehicle; Ani, anisodamine; Neo, neostigmine; CS, crush syndrome; WT, wild-type; KO, knockout; **P < 0.01 *vs*. Sham, ^##^P < 0.01 *vs*. CS + Veh.

In the compressed muscle tissue from C57BL/6 mice, crush injury decreased the levels of p-JAK2/JAK2 and p-STAT3/STAT3. Treatment with Ani/Neo combination (28 mg/kg Ani and 56 μg/kg Neo, i.p.) inhibited the decrease of p-JAK2/JAK2 and p-STAT3/STAT3. Methyllycaconitine (MLA, 10 mg/kg, i.p.), an α7nAChR antagonist, significantly inhibited the effects of Ani/Neo combination on p-JAK2 and p-STAT3 (Fig. 8A–C). In another set of experiments, crush injury induced a decrease of the p-JAK2/JAK2 and p-STAT3/STAT3 in both WT and α7nAChR KO mice. In wild-type mice, Ani/Neo combination significantly inhibited the decrease of the p-JAK2/JAK2 and p-STAT3/STAT3 in the compressed muscle tissue. However, in α7nAChR knockout mice, the combined treatment showed no effect on the decrease of pJAK2/JAK2 and pSTAT3/STAT3 induced by CS (Fig. 8D–F).

## Discussion

In this study, we for the first time demonstrated that administration of combined Ani and Neo could increase the survival rate in rat, rabbit and mouse CS models. Our results demonstrated that the best ratio of Ani to Neo to improve animal survival after CS was about 500:1 and the best doses of combination was 20 mg/kg Ani and 40 μg/kg Neo in rat CS models. Moreover, treatment with Ani/Neo combination not only improved MAP and SBP in rat CS models, but also decreased the levels of CK, CK-MB, BUN and Cr and K^+^ in serum in rat and rabbit CS models as well as the levels of H_2_O_2_, MPO and total NO in serum and compressed muscle in rat CS model. The protective effects of Ani/Neo combination on CS were attenuated in MLA pretreated CS mice and α7nAChR KO CS mice, suggesting that the beneficial effect of combined therapy on CS was at least partially mediated by the activation of α7nAChR.

It is noted that current therapeutic strategy for CS is mainly to improve symptoms including fluid administration to prevent shock and diuresis to prevent crush-related acute kidney injury. However, the mortality of CS is still at a high level and the treatment outcome is far away from satisfaction. As a result, more effective therapies are needed for CS patients, especially under emergency. Therefore, new therapies based on the underlying mechanisms of CS need to be developed.

Ani, extracted from a traditional Chinese herb *Scopolia taugutica*, acts as an antagonist for muscarinic cholinergic receptor, less toxic on CNS compared with atropine^9^,^12^. It was previously reported that Ani played an alleviative role in airway hyper-reactivity and inflammation in a mouse model of allergic asthma through the modulation of Th1/Th2 balance^12^. Several works from our laboratory have demonstrated the modulatory effects of Ani on inflammatory response in inflammatory and autoimmune diseases^9^,^13^. For example, we found that Ani significantly reduced the LPS-induced mortality and down-regulated levels of TNF-α and IL-1β through an α7nAChR-dependent signaling pathway in rats^9^. In addition, our work showed an alleviative effect of Ani on shock through an anti-inflammatory manner^13^. Those studies clearly demonstrated the anti-inflammatory effect of Ani. Recently, it has been shown that the combination of Ani with Neo augments the anti-inflammatory effect compared with Ani alone, since Neo, an inhibitor of acetylcholinesterase, can significantly increase the level of endogenous acetylcholine, thus enhancing the α7nAChR-mediated effects^11^,^14^,^15^. Combined treatment with Ani and Neo significantly attenuated the severity and symptoms of shock and collagen-induced arthritis^11^,^15^. In accordance with those studies, our current study showed that the administration of Ani/Neo combination enhanced the 24 h survival rate in rat, rabbit and mice CS models respectively with significantly improved MAP and SBP in rat CS model as well as decreased the levels of serum CK, CK-MB, BUN, Cr and K^+^ in rat and rabbit CS models. Those data show that the combining Ani and Neo is superior than Ani or Neo alone to protect animals with CS.

It has been reported that oxidative stress, including free radicals and other oxygen by-products, is elevated during CS especially after decompression, which leads to the subsequent damages to vital organs such as the heart and the kidney^16^. So far, several factors have been considered to contribute to the activation of oxidative stress under CS, including extensive muscle damage caused by squeezing, multiple organ failure, limb compression and mitochondria damage led to by the accumulation of muscle breakdown products^17^,^18^,^19^. As a result, inhibiting oxidative stress may play an important role in the treatment of CS. Here, in this work, we found that the administration of Ani/Neo combination (500:1) at the doses of 20 mg/kg in rat CS model at 30 min before decompression significantly decreased the levels of H_2_O_2_, MPO, total NO in serum and compressed muscle. These results suggest that combining application of Ani and Neo could alleviate the oxidative stress and thus contributes to improvement of survival after CS.

Traditionally, it is believed that anti-inflammatory response is mainly regulated by humoral factors^20^,^21^. However, recent studies indicated that neural regulation also participated in anti-inflammatory reaction^22^,^23^. Among all the neural regulatory pathways, “cholinergic anti-inflammatory pathway” was newly proposed by Tracey *et al*.^24^ as one of the major neural regulation mechanisms. They demonstrated that this pathway was mainly mediated by vagus nerve, which releases acetylcholine to act on various kinds of inflammatory cells and induces the production and secretion of pro-inflammatory cytokines. So far, several studies in out laboratory have demonstrated that the α7nAChR, a subtype of nicotinic acetylcholine receptor, is involved in the “cholinergic anti-inflammatory pathway”^11^,^25^,^26^,^27^. For example, it was reported by Fu *et al*. that targeting α7nAChR provided a potential therapeutic strategy in the treatment of cardio-cerebral-vascular diseases^25^. It was also demonstrated by Sun *et al*. that the combination of Ani and Neo produced an alleviative effect on shock mainly through targeting α7nAChR^11^. In our current study, we found that combined therapy decreased the levels of H_2_O_2_, MPO and total NO as well as the levels of TNFα, IL-6 and IL-10 in compressed muscle in WT mice with CS. The reduction of ROS production in inflammatory cells could contribute to the decrease of the production of pro-inflammatory factors. These effects of the Ani/Neo combination were attenuated in α7nAChR KO mice with CS, suggesting that α7nAChR is involved in the protective effect of combined therapy on CS.

In this study, we demonstrated the JAK2-STAT3 signaling pathway was activated by the administration of Ani/Neo combination via activation of α7nAChR, which played a significantly protective role in CS. Recent study by Fan *et al*. reported that administration of Ani contributed to the alleviation of CS in on-site mortality through the α7nAChR-mediated enhancement of insulin sensitivity and the increased phosphorylation of Na/K-ATPase in C2C12 myotubes, which led to the decrease of serum potassium^28^. We think this could be a mechanism for our observed beneficial effect of Ani/Neo combination on CS in this study. Furthermore, our study also showed that α7nAChR activation by the combined Ani/Neo application could produce anti-inflammation via JAK2-STAT3 activation^29^,^30^,^31^, which was attested by the reduction of the levels of TNFα and IL-6 in the crushed muscle. The anti-inflammation lead to better survival of skeletal muscle fibers during and after CS. It is consistent with the previous study in which the anti-inflammation effect of α7nAChR activation on skeletal muscle injury was also reported in muscular dystrophy^32^. In addition, Kami *et al*. reported that the activation of JAK2/STAT3 in skeletal fibers could also promote the survival of skeletal myocytes by enhancing the expression of anti-apoptotic proteins such as bcl-2 and bcl-xL^33^. We suspect that by all these α7nAChR-mediated JAK2/STAT3 activation effects, the combining use of Ani and Neo reduced the death of crushed skeletal muscle fibers (thus less K^+^ release from injured muscle), and enhanced K^+^ uptake by surviving skeletal muscles and eventually led to reduced serum K^+^, an important factor promoting the mortality of CS.

In conclusion, we for the first time show that the combining treatment of Ani and Neo at a ratio of 500:1 contributes to the alleviation of CS in animal models, increasing the 24 h survival rate and down-regulated the oxidative stress in muscle. This process is at least partially through the activation of α7nAChR-JAK2/STAT3 signaling. We believe that this study may provide a new way for the treatment of CS. The combining use of Ani and Neo has positive therapeutic effect on animal CS models, but there is a long way to go from bench to bedside before their side-effects, stability of the preparation, pharmacokinetics, efficacy and toxicology are determined.

## Methods

### Animals and agents

SD rats (250–270 g), C57BL/6 mice (25–30 g) and rabbits (2–3 kg) were obtained from Sino-British SIPPR/BK Laboratory Animal Ltd (Shanghai, China). α7nAChR knockout mice were acquired from Jackson Laboratory (B6.129S7-Chrna7tm1Bay, Stock Number: 003232, Bar Harbor, MA, USA). All animals were maintained under specific pathogen free conditions in the animal house at the Second Military Medical University and were fed with water and a standard diet ad libitum. All animal experiments were performed in accordance with the National Institute of Health Guide for the Care and Use of Laboratory Animals, and approved by the ethical committee for animal experiments of the Second Military Medical University. Ani was obtained from Shanghai First Biochemical Phamaceutical Company (Shanghai, China). Neo was from San-Wei Pharmaceutical Company (Shanghai, China). MLA was purchased from Sigma-Aldrich (St. Louis, MO, USA).

### CS model

CS models were generated as reported previously^5^,^34^,^35^. In brief, rats were fasted for 12 h and anesthetized with ketamine (10 mg/kg, i.p.) and diazepam (0.1 mg/kg, i.p.). After anesthesia, the rats were fixed in a self-made mold by scotch tape and a 20 kg iron block was placed on 4.5 cm above the ankle joint of hind-limbs for 5 h. Mice were fasted for 6 h and anesthetized with ketamine (15 mg/kg, i.p.) and diazepam (0.15 mg/kg, i.p.). After anesthesia, the mice were fixed in the self-made mold by scotch tape and a 20 kg iron block was placed on 2 cm above the ankle joint of hind-limbs for 4 h. For rabbits, 3% pentobarbital sodium (30 mg/kg, i.v.) was used for anesthesia and a 40 kg iron block was placed on 14 cm above the ankle joint of hind-limbs for 6 h.

### Blood pressure and heart rate measurements

Rats were anesthetized and then a polyethylene catheter was inserted into the common carotid artery for blood pressure measurement. Another catheter was inserted into external jugular vein for drug administration. The aortic catheter was connected to a transducer via a rotating swivel that allowed the animals to move freely in the cage. The blood pressure signal was digitized by a microcomputer. MAP, SBP and heart rate were determined in real time.

### CK, CK-MB, BUN, Cr and electrolyte measurements

Blood samples were collected at 6 h after decompression and allowed to clot for 2 h at room temperature before centrifugation for 20 min at 3000 g at 4 °C. The levels of CK, CK-MB, BUN, Cr and electrolyte in serum were measured using an Olympus AU640 autoanalyzer (Olympus, Hamburg, Germany) and adapted reagents from Olympus (Hamburg, Germany). Calibration and quality control of the machine were performed according to the instructions.

### H_2_O_2_ measurement

Levels of H_2_O_2_ in serum and muscle tissues were measured using a Hydrogen Peroxide Assay Kit (Beyotime, Shanghai, China). According to the instruction, samples were added with reaction reagent at room temperature for 30 min and measured immediately with a spectrophotometer at a wavelength of 560 nm. The concentration of H_2_O_2_ was calculated according to a standard curve generated with standard concentrations of H_2_O_2._

### MPO measurement

Levels of MPO in serum and muscle tissue were measured using an ELISA kit from R&D Systems (Minneapolis, MN, USA). In brief, 100 μl of the samples and standards were added in reagent diluent in 96-well microplate and then incubated 2 h at room temperature. After washing, 100 μl of detection antibody were added for 2 h at room temperature. After discarding the antibody, 100 μl of working dilution were added to each well for 20 min at room temperature. Then, after washing for 3X, 100 μl of substrate solution was added to each well and incubated for 20 min at room temperature. At last, added 50 μl of stop solution to each well. The absorbance was measured with a spectrophotometer at a wavelength of 540 nm, and concentrations of MPO were determined from standard curve.

### Total NO assay

Nitrate/nitrite was quantified by measuring the accumulation of nitrate. The standard solutions or samples were reacted with nitrate reductase for 40 min, and then Griess reagent I and Griess reagent II were added. After incubating for 10 min at room temperature, the absorbance was measured with a spectrophotometer at a wavelength of 540 nm, and concentrations of NO were determined from standard curve.

### Determination of TNFα, IL-6 and IL-10

Levels of TNFα, IL-6 and IL-10 in muscle tissue were measured using commercial enzyme-linked immunosorbent assay (ELISA) kits (Westtang biological system, Shanghai, SH, China).

### Western blotting

Proteins were extracted from muscle tissue using a standard extraction reagent supplemented with protease inhibitors (Kangchen, Shanghai, China). Protein concentration was determined using a bicinchoninic acid method (Beyotime). The proteins were separated using SDS-PAGE and electrotransferred to nitrocellulose membranes and then incubated with a primary antibody: p-JAK2 (Santa Cruz Biotechnology, Dallas, TX, USA, 1:500), JAK2 (Santa Cruz Biotechnology, Dallas, TX, USA, 1:500), p-STAT3 (Santa Cruz Biotechnology, Dallas, TX, USA, 1:500), STAT3 (Santa Cruz Biotechnology, Dallas, TX, USA, 1:500) for 8–12 h at 4 °C. Samples were then incubated with an IRDye800CW-conjugated secondary antibody (Rockland, Gilbertsville, PA, USA) for 1 h at 25 °C. The image was acquired with an Odyssey infrared imaging system (Li-Cor Bioscience, Lincoln, NE, USA). All immunoblotting experiments were repeated at least three times^36^,^37^.

### Statistical analysis

Data are presented as mean  ±  SD. For survival time analysis, Kaplan-Meier analysis was used, a Cox regression test following. Serial data of MAP, SBP was analysed with two-way analysis of variance (ANOVA) of repeated measures. For experiments involving only 1 factor, one-way ANOVA was used, followed by Student *t* test. Statistical significance was set at P < 0.05.

### Experimental Protocols

#### Effects of combined Ani and Neo on 24 h survival rate of rats with CS

The first set of experiments was performed to determine the best combination(s) of Ani and Neo to improve animal survival after CS. Rats were subjected to hind-limbs compression for 5 h, and then were injected with vehicle, Ani (0, 5, 10, and 20 mg/kg, i.p.), Neo (0, 5, 10, 20, and 40 μg/kg, i.p.), or combined Ani and Neo (n = 15 per group) at 30 min before decompression. In the second set of experiments, we aimed to determine the best doses of the Ani and Neo to improve animal survival after CS while the ratio of Ani to Neo was fixed at the best ratio determined in the 1^st^ set of experiments. The rats were subjected to hind-limbs compression for 5 h, and then they were treated with vehicle, Ani (20 mg/kg, i.p.), Neo (40 μg/kg, i.p.), and three Ani/Neo combinations (5 mg/kg and 10 μg/kg, 10 mg/kg and 20 μg/kg, 20 mg/kg and 40 μg/kg, i.p.) (n = 15 per group) at 30 min before decompression. In the third set of experiments, we were trying to determine the optimal time to give the best Ani/Neo combination discovered in the 2^st^ set of experiments. Rats were subjected to hind-limbs compression for 5 h, followed by vehicle and combined Ani/Neo (20 mg/kg and 40 μg/kg, i.p.) (n = 20 per group) at 30 min before decompression, immediately after decompression and 30 min after decompression respectively. Survival rate was determined within 24 h while animals were housed in the standard animal room (Room temperature: 22 °C; a 12-h light/dark cycle) with water and standard diet.

#### Effects of the Ani/Neo compound on MAP, SBP and heart rate in rats with CS

At 30 min after collection of baseline blood pressure and heart rate, rats were subjected to hind-limbs compression for 5 h followed by vehicle and the Ani/Neo compound (20 mg/kg Ani and 40 μg/kg Neo, i.p.) (n = 20 per group) at 30 min before decompression. MAP, SBP and heart rate were calculated over the first 4 h.

#### Effects of combined Ani and Neo at 500:1 ratio on CK, CK-MB, BUN, Cr and electrolyte in serum in rats with CS

Rats were subjected to hind-limbs compression for 5 h, followed by vehicle and three different doses of combined Ani/Neo (5 mg/kg and 10 μg/kg, 10 mg/kg and 20 μg/kg, 20 mg/kg and 40 μg/kg, i.p.) (n = 20 per group) at 30 min before decompression. 6 h after decompression, blood samples were collected. The levels of CK, CK-MB, BUN, Cr and electrolyte in serum were measured as mentioned above.

#### Effects of the Ani/Neo compound on H_2_O_2_, MPO and NO in serum and compressed muscle in rats with CS

Rats were subjected to hind-limbs compression for 5 h, and then injected (i.p.) with vehicle, Ani (20 mg/kg, i.p.), Neo (40 μg/kg, i.p.) or a combination of 20 mg/kg Ani and 40 μg/kg Neo (i.p.) at 30 min before decompression (n = 10 per group). Blood samples and compressed muscle tissue were collected at 6 h after decompression. The level of H_2_O_2_, MPO, total NO in serum and compressed muscle were determined as mentioned above.

#### Effects of combined Ani and Neo at 500:1 ratio on 24 h survival rate in rabbits with CS

Rabbits were subjected to hind-limbs compression for 6 h, followed by vehicle and three different doses of combined Ani/Neo (2.5 mg/kg and 5 μg/kg, 5 mg/kg and 10 μg/kg, 10 mg/kg and 20 μg/kg, i.p.) (n = 16 per group) at 30 min before decompression. Survival rate was determined for the following 24 h.

#### Effects of combined Ani and Neo at 500:1 ratio on CK, CK-MB, BUN, Cr and K^+^ in serum in rabbits with CS

Rabbits were subjected to hind-limbs compression for 6 h, followed by vehicle and three different doses of combined Ani/Neo (2.5 mg/kg and 5 μg/kg, 5 mg/kg and 10 μg/kg, 10 mg/kg and 20 μg/kg, i.p.) (n = 8 per group) for 30 min before decompression. Blood samples were collected at 6 h after decompression. The levels of CK, CK-MB, BUN, Cr and K^+^ were measured as mentioned above.

#### Effects of combined Ani and Neo at 500:1 ratio on 24 h survival rate in WT and α7nAChR KO mice with CS

α7nAChR KO mice and the corresponding WT controls were subjected to hind-limbs compression for 4 h, and then treated with vehicle, Ani (28 mg/kg, i.p.), Neo (56 μg/kg, i.p.) and a combination of 28 mg/kg Ani and 56 μg/kg Neo (i.p.) at 30 min before decompression. Survival rates were monitored for the ensuing 24 h.

#### Effects of combined Ani and Neo at 500:1 ratio on H_2_O_2_, MPO, NO, TNFα, IL-6 and IL-10 in compressed muscle in WT and α7nAChR KO mice with CS

WT and α7nAChR KO mice were subjected to hind-limbs compression for 4 h, and then treated with vehicle, Ani (28 mg/kg, i.p.), Neo (56 μg/kg, i.p.) and a combination of 28 mg/kg Ani and 56 μg/kg Neo (i.p.) at 30 min before decompression. Compressed muscles were collected at 6 h after decompression. Levels of H_2_O_2_, MPO, total NO, TNFα, IL-6, and IL-10 in compressed muscle were measured as mentioned above.

#### Effects of the combined Ani and Neo at 500:1 ratio on the phosphorylation of JAK2 and STAT3 in the compressed muscle tissue from C57BL/6 mice, WT and α7nAChR KO mice with CS

C57BL/6 mice were subjected to hind-limbs compression for 4 h and then were intraperitoneally injected with vehicle; combined Ani/Neo (28 mg/kg and 56 μg/kg, i.p); MLA (10 mg/kg, i.p.); combined Ani/Neo+ MLA (i.p.). In another set of experiments, WT and α7nAChR KO mice were subjected to hind-limbs compression for 4 h, followed by treatment with vehicle, or combined treatment (28 mg/kg Ani and 56 μg/kg Neo, i.p) at 30 min before decompression. Compressed muscles were collected at 6 h after decompression. The total and phosphorylation of JAK2 and STAT3 were measured as mentioned above.

## Additional Information

**How to cite this article**: Xu, Z.-Q. *et al*. Combined administration of anisodamine and neostigmine rescued acute lethal crush syndrome through a7nAChR-dependent JAK2-STAT3 signaling. *Sci. Rep*. **6**, 37709; doi: 10.1038/srep37709 (2016).

**Publisher’s note:** Springer Nature remains neutral with regard to jurisdictional claims in published maps and institutional affiliations.

## Figures and Tables

**Table 1:** Effects of Ani and Neo on 24 h survival rate in rat CS model.

Ani		Dose (mg/kg)			
Neo		0	5	10	20
Dose (μg/kg)	0	13.3	33.3	46.7	60.0**
	5	20.0	53.3*	60.0**	60.0**
	10	33.3	60.0**	66.7**	60.0**
	20	40.0	66.7**	80.0***	66.7**
	40	46.7**	60.0**	66.7**	86.7***

Rats were subjected to hind-limbs compression for 5 h, followed by treatment with vehicle, Ani (5, 10, and 20 mg/kg, i.p.), Neo (5, 10, 20 and 40 μg/kg, i.p.), or combination of Ani and Neo at 30 min before decompression. Survival rates were monitored for the ensuing 24 h. n = 15 per group. *P < 0.05 *vs*. vehicle, **P < 0.01 *vs*. vehicle, ***P < 0.001 *vs*. vehicle. Ani, anisodamine; Neo, neostigmine.

**Table 2:** Effects of combined Ani/Neo at 500:1 ratio on serum CK, CK-MB, BUN, Cr and K^+^ in rat CS model.

Group	CK (U/mL)	CK-MB (U/mL)	BUN (μmol/L)	Cr (μmol/L)	K^+^ (mmol/L)
Sham	0.7 ± 0.2	1.2 ± 0.5	8.0 ± 1.1	22.2 ± 2.6	6.5 ± 0.45
Vehicle	57.2 ± 15.2**	3.2 ± 1.2*	29.7 ± 2.0**	108 ± 20.5**	11.3 ± 0.7**
Ani(5 mg/kg)+ Neo(10 μg/kg)	44.3 ± 1.9^##^	2.5 ± 0.4^#^	28.2 ± 1.8^#^	90.2 ± 16.2^#^	10.1 ± 0.5^#^
Ani(10 mg/kg)+ Neo(20 μg/kg)	38.7 ± 6.9^##^	2.2 ± 0.3^##^	27.5 ± 2.3^##^	76.3 ± 21.9^##^	9.3 ± 0.4^##^
Ani(20 mg/kg)+ Neo(20 μg/kg)	36.1 ± 4.9^##^	2.6 ± 0.3^##^	26.4 ± 2.6^##^	59.7 ± 13.1^##^	9.1 ± 0.5^##^

Rats were subjected to hind-limbs compression for 5 h, followed by treatment with vehicle or three different doses of Ani/Neo combination with a ratio of 500:1 at 30 min before decompression. Blood samples were collected at 6 h after decompression. Levels of CK, CK-MB, BUN, Cr and K^+^ in serum were measured. n = 20 per group. *P < 0.05 *vs*. Sham, **P < 0.01 *vs*. Sham, ^#^P < 0.05 *vs*. vehicle, ^##^P < 0.01 *vs*. vehicle. Ani, anisodamine; Neo, neostigmine.

**Table 3:** Effects of combined Ani and Neo at 500:1 ratio on serum CK, CK-MB, BUN, Cr and K^+^ levels rabbit CS model.

Group	CK (U/mL)	CK-MB (U/mL)	BUN (μmol/L)	Cr (μmol/L)	K^+^ (mmol/L)
Sham	2.3 ± 1.93	0.41 ± 0.15	19.1 ± 1.83	101 ± 17.6	5.38 ± 0.58
Vehicle	128.4 ± 15**	2.97 ± 0.49**	23.9 ± 2.31*	133 ± 29.9*	12.0 ± 1.2**
Ani(2.5 mg/kg)+ Neo(5 μg/kg)	124.6 ± 16.7	2.74 ± 0.53	23.1 ± 2.31	116 ± 31.9	10.5 ± 1.99^#^
Ani(5 mg/kg)+ Neo(10 μg/kg)	93.2 ± 17.1^##^	2.62 ± 0.54^#^	22.8 ± 2.06	111 ± 19.1	10.4 ± 1.27^#^
Ani(10 mg/kg)+ Neo(20 μg/kg)	91.5 ± 9.55^##^	2.47 ± 0.25^#^	19.9 ± 2.96^##^	98.9 ± 22.7^#^	9.22 ± 1.12^##^

Rabbits were subjected to hind-limbs compression for 6 h, followed by treatment with vehicle or Ani/Neo combination with a ratio of 500:1 at 30 min before decompression. Blood samples were collected at 6 h after decompression. Levels of CK, CK-MB, BUN, Cr and K^+^ in serum were measured. n = 8 per group. *P < 0.05 *vs*. Sham, **P < 0.01 *vs*. Sham, ^#^P < 0.05 *vs*. vehicle, ^##^P < 0.01 *vs*. vehicle. Ani, anisodamine; Neo, neostigmine.
